# Role of actor networks in primary health care implementation in low- and middle-income countries: a scoping review

**DOI:** 10.1080/16549716.2023.2206684

**Published:** 2023-05-03

**Authors:** Dominic Dormenyo Gadeka, Patricia Akweongo, Eleanor Whyle, Genevieve Cecilia Aryeetey, Justice Moses Aheto, Lucy Gilson

**Affiliations:** a Department of Health Policy, Planning and Management, University of Ghana School of Public Health, Legon-Accra, Ghana; b Division of Health Policy and Systems, School of Public Health and Family Medicine, University of Cape Town, Cape Town, South Africa; c Department of Biostatistics, University of Ghana School of Public Health, Legon-Accra, Ghana; d Department of Global Health and Development, London School of Hygiene and Tropical Medicine, London, UK

**Keywords:** Policy implementation, primary health care, actor networks, social network analysis, health policy analysis

## Abstract

**Background:**

Primary health care (PHC) improvement is often undermined by implementation gaps in low- and middle-income countries (LMICs). The influence that actor networks might have on the implementation has received little attention up to this point.

**Objective:**

This study sought to offer insights about actor networks and how they support PHC implementation in LMICs.

**Methods:**

We reviewed primary studies that utilised social network analysis (SNA) to determine actor networks and their influence on aspects of PHC in LMICs following the five-stage scoping review methodological framework by Arksey and O’Malley. Narrative synthesis was applied to describe the included studies and the results.

**Results:**

Thirteen primary studies were found eligible for this review. Ten network types were identified from the included papers across different contexts and actors: professional advice networks, peer networks, support/supervisory networks, friendship networks, referral networks, community health committee (CHC) networks, inter-sectoral collaboration networks, partnership networks, communications networks, and inter-organisational network. The networks were found to support PHC implementation at patient/household or community-level, health facility-level and multi-partner networks that work across levels. The study demonstrates that: (1) patient/household or community-level networks promote early health-seeking, continuity of care and inclusiveness by enabling network members (actors) the support that ensures access to PHC services, (2) health facility-level networks enable collaboration among PHC staff and also ensure the building of social capital that enhances accountability and access to community health services, and (3) multi-partner networks that work across levels promote implementation by facilitating information and resource sharing, high professional trust and effective communication among actors.

**Conclusion:**

This body of literature reviewed suggests that, actor networks exist across different levels and that they make a difference in PHC implementation. Social Network Analysis may be a useful approach to health policy analysis (HPA) on implementation.

## Introduction

Primary health care (PHC) improvement is often undermined by implementation gaps in low- and middle-income countries (LMICs). Pursuant to the Alma Ata declaration on PHC, which enjoined nations to make health care accessible, affordable, and situated in the cultural context of the people [1], LMICs adopted bottom-up approaches towards achieving the PHC goal [2,3]. Experience since the declaration has shown PHC as an important strategy in improving both population health and in making healthcare systems more effective, responsive and efficient [4]. Evidence further shows PHC as a cost-effective strategy and an essential tool for achieving quality Universal Health Coverage (UHC) and the health-related Sustainable Development Goals (SDGs) [4–7]. However, PHC implementation in LMICs remains poor [4]. Efforts to address the gap often focus on new ways of delivering services or extending coverage [8,9].

Bottom-up theory suggests that networks play an important role in policy implementation [10,11]. This body of theory emphasises the discretionary power and critical influence of ‘policy implementors’ over policy implementation, even as top-down implementation is the norm of practice in many settings [12]. For instance, bottom-up theorists Hanf and colleagues studied the goals of actors in an intervention delivery process, and concluded that policy implementation through networks (such as networks of frontline service providers) are more successful than implementation carried out through a top-down approach [10]. Additionally, Elmore [13] emphasises that bottom-up implementors possess widely dispersed informal power that are critical for problem-solving and which promote success or otherwise of a policy implementation. However, the influence that actor networks might have on implementation has received little attention up to this point.

Bottom-up theory further argues that in order to strengthen implementation, the goals, strategies, activities, and contacts of actors involved in the implementation process must be understood. Furthermore, earlier work that mapped existing body of health policy implementation research in lower-income settings specifically highlights the need to consider the nature and role of actor networks in understanding why and how implementation varies across policy types [14]. However, implementation research in LMICs is scarce, and actor-network-focused research is rare. Understanding how networks contribute to policy implementation, particularly, PHC in LMICs may help to strengthen its implementation. Strengthening PHC implementation requires working through many sets of actors, but it is not clear whether, which and how networks among implementors support implementation. In recent years, Social Network Analysis (SNA) has begun to be applied in health policy analysis (HPA) work. Such analysis can deepen understanding of who forms networks and how networks function. These insights could be useful in understanding how networks influence policy change, including PHC implementation.

This review offers insights about actor networks and how they support PHC implementation in LMICs. It further provides evidence on the usefulness of SNA in HPA in understanding how networks support policy implementation and research gaps.

## Methods

The study adopted a scoping review approach to understand the roles actor networks play in PHC implementation, specifically in LMICs and to consider the implications for policy and practice. Our study therefore focused on all sources of information from full range of literature available on actor networks in PHC implementation in LMICs. The study followed the five-stage scoping review methodological framework by Arksey and O’Malley [15] as updated by Levac, Colquhoun, and O’Brien [16]: (1) identifying the research question (2) identifying relevant studies (3) study selection (4) charting the data and (5) collating, summarising and reporting the results. In accordance with the standard approach to conducting scoping reviews, a quality appraisal was not performed. However, the Preferred Reporting Item for Systematic Reviews and Meta-Analyses extension for Scoping Reviews (PRISMA-ScR) [17] criteria was used to guide the conduct and reporting of the review. Ethical approval or patient consent was not required.

### Identifying the research question

The specific scoping review question is ‘What role do actor networks play in PHC implementation and how do networks add to our understanding of implementation processes in low-and-middle-income countries’?

### Identifying relevant studies

We attempted to identify all relevant scholarly primary research studies conducted in LMICs and reported in the English language and with no date limits. We did not include unpublished studies in order to ensure quality of the findings of the review. The date limit was left open to enable the collection of all relevant articles to ensure richness of data to answer the research question. Electronic searches of public health, social science, and medical peer-reviewed journals were carried out in PubMed, Sociological Abstracts, Social Science Research Network (SSRN), and PsychINFO databases using logical operator-based combinations of key terms. The initial search used a broad search strategy involving free-text terms, synonyms and subject headings relating to actor networks, social networks and PHC. The search strategy consisted of the main terms, actor networks, social networks, social network analysis, while the subject headings consisted of role of social networks in PHC, role of actor networks in PHC implementation and influence of social networks on PHC in low- and middle-income countries. Finally, a general search combining the terms ‘social network’ or ‘social network analysis’ or ‘actor networks’ and ‘primary health care’ and ‘community health workers,’ ‘community health committees,’ ‘health promotion,’ ‘disease prevention,’ ‘rehabilitation,’ ‘palliative care,’ ‘health financing’ ‘health service provision’ ‘health workers’ health information” ‘medicines and technology’ ‘health systems governance and leadership’ or ‘low- and middle-income country,’ was completed. We did not use the term ‘implementation’ in our search strategy because most authors who utilised SNA as an approach already focused on implementation. Reference lists of retrieved primary articles were checked for further potentially relevant studies that may have been missed by the electronic search. The website of the International Network for Social Network Analysis (www.insna.org), including linked sites and contents were hand-searched. We further hand-searched the journals *Social Networks* and *Implementation Science* to identify additional studies.

### Study selection

To be eligible for the review, primary studies had to report on the relationship between networks (actor networks or social networks) and any aspect of PHC implementation in LMICs. The study selection was an iterative process. It involved searching the literature, refining the search strategy and reviewing articles for inclusion [16]. To be included, studies had to report use of SNA in the design of the study, for example, ‘net-map’, social network mapping, assessment of network structure and properties. Limiting the study to papers using SNA ensured that studies formally considered networks as the central elements of their analysis. We included quantitative, qualitative and mixed-method studies. All studies that met the inclusion criteria were exported to Mendeley, with duplicates identified and removed.

Studies were excluded if social networks were mentioned but the type of analysis was only descriptive, that is, without the determination of the role or influence of the social networks on PHC. Also, studies were excluded if the type of analysis was not reported although the study involved a social network. Additionally, social network studies published in languages other than English language were excluded. Furthermore, where studies reported the same data in multiple papers, only one paper was included to avoid duplication.

### Charting the data

We developed a data charting form in Microsoft Excel. The data extracted included study title, study objective, authors, year of publication, the country, study design/methods, study population, networks reported and key findings that related to the review question. The first author performed the data charting and was reviewed and doubled checked by all the other authors. We resolved all disagreements through discussions until reaching consensus. We discussed the results and continuously updated the data charting form in an iterative process.

### Collating, summarising, and reporting the results

We applied a narrative synthesis to present the results of the included studies following recommendation by Levac et al. [16]. We collated and compared definitions of network(s) provided by the authors in each paper to observe any commonalities or differences. The networks were then classified into types based on the definitions drawn from the reviewed papers for each of the identified networks. Through an inductive process, we extracted, coded and categorised evidence from the included articles to identify the roles played by the different network types. To understand how the networks support PHC implementation, we further categorised the evidence from the included articles based on the three inter-related and synergistic components of PHC: (1) individual, households/families and community empowerment for increased social participation and enhanced self-care and self-reliance in health (2) comprehensive integrated health services that embrace primary care as public health goods and (3) multi-sectoral policies and actions to address wider determinants of health [7].

## Results

In this section, we will first present the results on the extent, range, and nature of research activity on actor networks in PHC implementation in LMICs, then discuss the various types of actor networks identified in the literature and then the different roles played by the different network types and how they support PHC implementation in the following sections.

**Figure 1.**
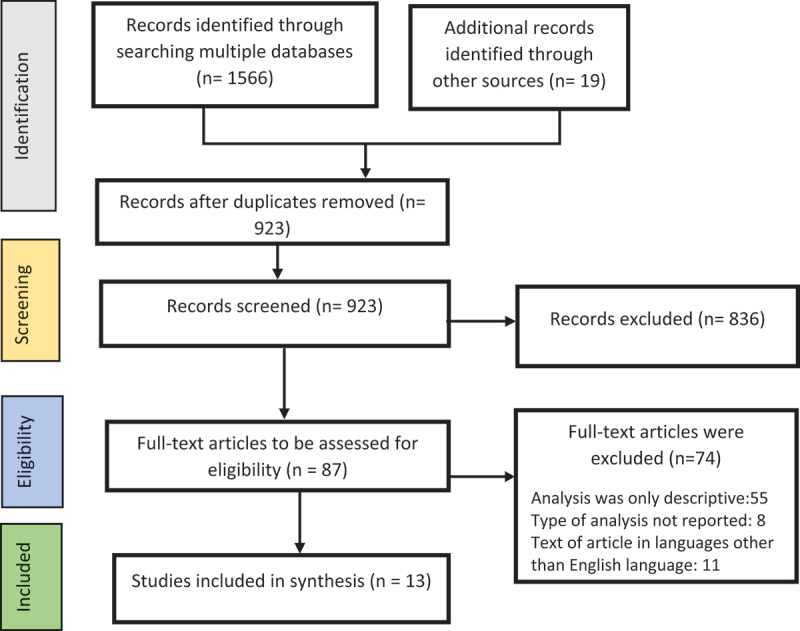
Flow diagram of study selection process.

Figure 1 presents a flow diagram of the selection process including the total studies identified, the excluded studies, reasons for exclusion and the final included studies. A total of 923 potentially relevant studies were identified from searches of electronic databases and review article references after the removal of duplicates. After title and abstract screening, 836 were excluded because the studies only mentioned social network analysis without applying it to their work. The remaining 87 full-text articles were retrieved and assessed for eligibility. We noted that most of the studies identified were descriptive in nature and do not systematically examine the relationships that exist between networks and PHC. Based on this, we excluded 74 out of the 87 articles for only providing a descriptive view of SNA, thus without the determination of the role or influence of social networks on PHC. The remaining 13 studies were considered eligible for this review.

### Extent, range, and nature of research activity on actor networks in PHC implementation in LMICs

Table 1 presents the full list of the included studies in this review. The different papers examined different networks, with each paper primarily examining one type of network. Together, the articles reported on PHC implementation using SNA from nine different countries. Most studies were reported from Tanzania (*n* = 3) [18–20], followed by South Africa (*n* = 2) [21,22] and India (*n* = 2) [23,24]. The articles were largely recent with publication dates ranging from 2014 to 2020. The highest number of publications were seen in 2019 [21,23–26] and 2020 [18,19,27,28]. This indicates the recent adoption of SNA in PHC research in LMICs. Most of the studies (*n* = 11) applied cross-sectional design [18,19,21–23,25–30]. All the studies applied a quantitative social network data collection method, while six of these applied a mixed methods approach [22,23,25–28]. The qualitative approaches used ranged from in-depth interviews, focus group discussions, observation and document review. One article reports on the pre-test post-test social network analysis approach [24], while another was conducted as part of a cluster-randomised HIV prevention trial [20]. Study populations were diverse, ranging from community members [19,20,24,26,30] to district health managers [22,29] and actors beyond the district level [25,27] who influence PHC implementation. There were considerable variations in the areas of PHC addressed in each set of articles. These include supervisory relationships of community health workers in PHC [21]; health behaviours and self-reported health [30] patient referrals [18]; inter-sectoral collaboration for people-centred mental health care [25]; community health committees in rural and urban settings [26]; antenatal care utilisation [19]; HIV risk behaviour and normative beliefs [20]; co-ordination between women’s self-help groups and local health systems [24]; professional advice for PHC workers [28]; vaccine delivery [27]; organisational infrastructure for service delivery [29] and communication between disease programmes and district managers [22].

**Table 1.** Overview of the included papers.

Authors (Year)	Study title	Country	Method/Study design	Study Population
Assegaai & Schneider (2019)	The supervisory relationships of community health workers in primary health care: social network analysis of ward- based outreach teams in Ngaka Modiri Molema District, South Africa	South Africa	Cross-sectional study design with quantitative social network analysis (SNA) approach	Community Health Workers (CHWs), ward-based outreach team (WBOT), PHC facility managers and local area managers
Chami, Ahnert, Voors, & Kontoleon, (2014)	Social Network Analysis Predicts Health Behaviours and Self-Reported Health in African Villages	Liberia	Cross-sectional study design with quantitative household survey SNA approach	Households in highly remote, post-conflict villages in Liberia
Francetic, Tediosi, & Kuwawenaruwa (2020)	A network analysis of patient referrals in two district health systems in Tanzania	Tanzania	Cross-sectional study design with quantitative facility-based SNA approach	Public health facilities in the districts
Hall et al. (2019)	Intersectoral collaboration for people‑centred mental health care in Timor‑Leste: a mixed‑methods study using qualitative and social network analysis	Timor‑Leste	Cross-sectional study design with a mixed method SNA approach	Key stakeholders from government, NGO, civil society and other organisations working in national mental health and social care
Hoe et al. (2019)	Using social network analysis to plan, promote and monitor intersectoral collaboration for health in rural India	India	Cross-sectional study design with mixed method SNA approach	Key informants from organisations working on MCH and/or WASH in Kachhauna, Uttar Pradesh, India.
Karuga et al. (2019)	“It’s like these CHCs don’t exist, are they featured anywhere?”: Social network analysis of community health committees in a rural and urban setting in Kenya	Kenya	Cross-sectional study design with a mixed method SNA approach	Community discussants and health professionals in a rural area and an urban slum
Mukong & Burns (2020)	Social networks and antenatal care utilisation in Tanzania	Tanzania	Cross-sectional study design with quantitative SNA approach	Women aged 15–49 years.
Mulawa et al. (2016)	Evidence of social network influence on multiple HIV risk behaviors and normative beliefs among young Tanzanian men	Tanzania	Cluster-randomised HIV prevention trial with quantitative SNA approach	Urban social networks locally referred to as “camps”.
Ruducha et al., (2019)	Measuring coordination between women’s self-help groups and local health systems in rural India: A social network analysis	India	Pretest, post-test design with quantitative SNA approach	Self-help groups (SHG) members, village-level and block-level government health workers, and other key members of the community
Sabot et al., (2020)	Professional advice for primary healthcare workers in Ethiopia: A social network analysis	Ethiopia	Observational, cross-sectional study design with quantitative SNA approach	Staff at primary healthcare units
Soi et al., (2020)	Global health systems partnerships: A mixed method analysis of Mozambique’s HPV vaccine delivery network actors	Mozambique	Cross-sectional study design with a mixed-method SNA approach	Frontline Ministry of Health workers, Ministry of Education staff and supporting partner organisation members
Ssengooba, Kawooya, Namakula, & Fustukian, (2017)	Application of social network analysis in the assessment of organisation infrastructure for service delivery: A three district case study from post-conflict northern Uganda	Uganda	Cross-sectional study design with a mixed method SNA approach	District Health Offices (DHOs) and service provider organisations (SPOs)
Kawonga, Blaauw, & Fonn, (2015)	Exploring the use of social network analysis to measure communication between disease programme and district managers at sub-national level in South Africa	South Africa	Cross-sectional study design with quantitative and qualitative SNA approach	Health managers located at subnational level

### Actor networks identified to support PHC implementation in LMICs

Ten types of actor networks across different contexts and actors were identified in the pool of papers we reviewed (Table 2). The network types include professional advice networks [28], peer networks [19,20], support/supervisory networks [21], friendship networks [30], referral networks [18,24], community health committee (CHC) networks [26], inter-sectoral collaboration networks [23,25], partnership networks [27], communications networks [22] and inter-organisational networks [29]. The definitions for each of the network types are provided in Table 2. We also provided the descriptions drawn from the reviewed papers for each of the identified networks. Different terms were used to denote the meanings of the different network types based on the different contexts and the network goal. For instance, friendship [30] and peer networks [19] were reported to represent two different network types by the different authors based on the goal of the network (Table 2). Similarly, referral networks, which were examined in two of the reviewed articles [18,24] were reported with different meanings based on the different contexts.

**Table 2.** Actor networks and their definitions.

Network type	Network definition	Network description from reviewed articles
Friendship network	Close individuals who comfortably help each other on a given issue	Close friends who do not live in the same household but are comfortable to either turn to each other for advice, interest-free loan, or ask for help with harvest without paying [30].
Referral network	Relations between two or more health facilities involved in the sharing of patients from lower to higher levels of care	Networks where each referral represents a directional tie between a pair of health facilities [22].
Inter-sectoral collaboration network	Sets of organisations from different sectors which interact to achieve a particular objective	Any planning, information and resource sharing for a common purpose between organisations from different sectors and/or across thematic areas [24,29].
Partnership network	Sets of actors or agencies that formally or informally engage for a common purpose	A collaboration with the mission of accomplishing a common goal either contractually or non-contractually [27].
Community health committee networks	Group of individuals who serve as liaisons between community members and frontline health workers in the provision of health services to the community	A group of community-based committees who provide and received health-related information and are able to participate in the exchange of information and decision-making on community health services [26].
Support/supervisory networks	Two or more actors who provide support to one another based on hierarchies of the health system	A set of relationships embedded in the wider context of social and professional relationships and hierarchies within the health system [20].
Peer networks	Group or individuals with similar sociodemographic categorisation and who get along either formally, informally or both and interact on a given issue	Semiformal groups who are either friends, acquaintances, or people who get along and/or socialise regularly [19,20].
Inter-organisational network	Network formed by two or more organisations with the purpose of achieving a common goal	Relational architecture or networks among organisations to strengthening the health workforce in post-conflict northern Uganda or any linkage [support/activity/engagement] between the respondent organisation and another external organisation for strengthening health workforce [29.]
Communications networks	Communication relationship that facilitates action or decision-making among individuals or organisations	Communications involving managers through one-on-one task-related communication or communication through co-participating in management committees [22].
Professional advice networks	Individuals or group of healthcare professionals who share advice on a common issue of interest	Healthcare professionals, either having provided or sought advice for a common objective or policy of interest [28].

### How networks support PHC implementation in LMICs

We identified that although the reviewed articles focused on specific networks, they also provide evidence on the wider context within which the networks were based. In line with the three inter-related and synergistic components of PHC [7], the review showed (Tables 3 and 4) that PHC-linked networks are engaged at the patient/household/community level [19,20,26,30], health facility-level (networks that support health care providers) [18,21,24,28]; the review also revealed multi-partner networks that work across levels [22,23,25,27,29].

**Table 3.** Findings on networks among implementors.

Identified actor networks	Authors (Year)	Study objective	Key findings
Professional advice networks	Sabot et al., (2020)	To compare professional advice networks of healthcare workers in eight primary healthcare units across four regions of Ethiopia	The study established that primary health care unit staff involved in the delivery of maternal and newborn health services have informal advice networks outside of supervisory structures. These advice networks of the healthcare workers in PHC units were observed to provide better change in health provider practices compared to when health care workers we-re only given training (example, in-service training) without the involvement of their networks.
Peer networks	Mukong & Burns, (2020)	To examine the impact of information externalities generated through network membership on antenatal care utilisation in Tanzania	Peer networks were seen to increase the probability of early antenatal check-up and antenatal completion. The study further found that relying on strong peer network increases the probability of antenatal completion by 23% and early antenatal initiation by 48%.
	Mulawa et al., (2016)	The study sought to evaluate the effectiveness of a camp-randomised Microfinance and health leadership intervention on sexually transmitted infections, gender-based violence and HIV risk behaviors	It was observed that peer networks of young men explained between 5.78 and 7.17% of variance in men’s normative beliefs and between 1.93 and 15.79% of variance in HIV risk behaviors
Support/supervisory networks	Assegaai & Schneider, (2019)	To identify critical actors and patterns of relationships in the supervision of ward-based outreach teams (WBOT) in a rural South African district	Support/supervisory networks were found to enable the WBOTs in drawing on sympathetic cadres identified among the PHC facility staff for support. Additionally, supervisory relationships within networks (teams) were observed to function better than those between teams and the rest of the PHC system
Friendship networks	Chami, Ahnert, Voors, & Kontoleon, (2014)	To predict health outcomes and to explain the susceptibility of a household to partake in health interventions in impoverished and under-researched developing country contexts	The findings show that friendship network helps in determining the number of ill family members, whereas the closeness of the members of the friendship network determines the individuals’ engagement in preventative health. Also was found to influence the individuals’ self-reported health status. The study further shows that the closeness between members in a friendship network predicts susceptibility to, instead of influence over, good health behaviours
Referral networks	Francetic, Tediosi, & Kuwawenaruwa, (2020)	To explore patient referral networks in two rural districts in Tanzania, Kilolo and Msalala	It was shown that referral networks facilitate the sharing of patients among health facilities
	Ruducha et al., (2019)	To assess how the health coordination and emergency referral networks between women’s self-help groups (SHGs) and local health systems have changed over the course of a 2-year learning phase of the Uttar Pradesh Community Mobilisation Project, India.	It was observed that the referral networks expanded relationships at the village level. It was established that the new relationships between the traditionally under-represented communities and local government could serve as vehicle for building social capital that could lead to a more accountable and accessible community health delivery system
Community Health Committee (CHC) networks	Karuga et al., (2019)	To explore the structure of a rural and an urban Community Health Committee (CHC) network and to analyze how health-related information flowed in these networks	CHC networks served as back-ups to government in addressing primary health care challenges by supporting Chiefs to implement government directives, such as compulsory child immunisation and discouraging banned cultural practices.
Inter-sectoral collaboration networks	Hall et al., (2019)	To investigate intersectoral collaboration for people-centred mental health care in Timor-Leste, a South-East Asian country in the process of strengthening its mental health system	Intersectoral collaboration networks facilitate information and resource sharing among organisations working within the health and social (disability and violence support) sectors in Timor-Leste even in resource restricted settings. It further revealed that contrary to the assumption that mental health services and system strengthening are led by the Ministry of Health, intersectoral collaboration networks resulted in split in stewardship for mental health between subnetworks in the health and social sectors
	Hoe et al., (2019)	To understand intersectoral collaboration between the organisations working on maternal & child health (MCH) and water & sanitation (WASH) before and immediately after the implementation of the HCL Foundation (HCLF)-funded HCL Samuday Project (2015–2017) in a rural block of Uttar Pradesh state, India	Intersectoral collaboration networks enabled growth of the network of organisations working on the MCH and WASH. Additionally, intersectoral collaboration networks were able to enhance organisations’ states in serving as gatekeepers of information and also enhanced their ability to play a coordinator role
Partnership networks	Soi et al., (2020)	To examine Mozambique’s Gavi driven partnership network which delivered human papillomavirus (HPV) vaccine during the demonstration phase	Partnership networks enabled high professional trust. Also, the composition and practices of the partnership resulted in complementary relationship that engendered a favorable collaborative environment in which the best that each institution had to offer was leveraged
Communication networks	Kawonga, Blaauw, & Fonn, (2015)	To explore the use of social network analysis (SNA) to measure communication between disease programme and district managers at sub-national level in South Africa	Communication networks between the actors involved enhance the flow of information among provincial programme managers. The study further shows that communication networks facilitate complex and varied interactions, including both collaborative as well as siloed communication amongst programme and district actors
Inter-organisational networks	Ssengooba, Kawooya, Namakula, & Fustukian, (2017)	To assess the inter-organisation infrastructure that supports the provision of selected health services in the reconstruction phase after conflict in northern Uganda	Inter-organisational networks enabled organisations to exhibit a broad range of functional roles in supporting the provision of the services. It further shows that inter-organisational networks present a better opportunity for organisations to leverage for faster communication and resource flow to boost the delivery of health services

**Table 4.** Summary of how networks support implementation.

Networks	Authors (Year)	How networks support PHC implementation
Patient/household/community-level networks	Mukong & Burns, (2020); Mulawa et al., (2016); Chami, Ahnert, Voors, & Kontoleon, (2014); Karuga et al., (2019)	Increase the probability of early antenatal check-up and antenatal completion Ensures alignment in good behavioral practices at the community level Enables the engagement of community members in preventative health. Help in predicting the susceptibility of individuals to good health behaviours Enables local implementers such as Chiefs to implement government directives
*Health facility-level networks*	Sabot et al., (2020); Ruducha et al., (2019); Francetic, Tediosi, & Kuwawenaruwa, (2020); Assegaai & Schneider, (2019)	Enable the provision of better change in health provider practices Helps health workers in drawing on sympathetic cadres identified among the PHC facility staff for support Facilitate the sharing of patients among health facilities Helps in building social capital that could lead to a more accountable and accessible community health delivery system
Multi-partner networks that work across levels	Hall et al., (2019); Hoe et al., (2019); Soi et al., (2020); Kawonga, Blaauw, & Fonn, (2015); Ssengooba, Kawooya, Namakula, & Fustukian, (2017)	Facilitate information and resource sharing among organisations Enabled growth of the network of organisations serving as gatekeepers of information Enabled high professional trust among partners/stakeholders Facilitate complex and varied interactions among stakeholders Enable organisations to leverage for faster communication and resource flow to boost the delivery of health services

### Patient/household/community-level networks

The study demonstrated that patient/household or community-level networks promote early health-seeking, continuity of care and inclusiveness by enabling network members (actors) the support that ensures access to PHC services (Table 4). The synthesis showed that patient/household or community-level networks vary considerably in their specific application and level of support for PHC (Tables 3 and 4). Four patient/household or community-level network types were identified in the reviewed articles [19,20,26,30]. Two were peer networks among health care users [19,20] while the rest were friendship [30] and community health committee (CHC) networks [26]. With regard to the peer networks, it was noted that pregnant women leveraged their contacts and sources of information externalities regarding antenatal care utilisation to increase their probability of early antenatal check-up and antenatal completion [19]. Similarly, young Tanzanian men used their peer networks to support individuals’ engagement in preventative health in the prevention of multiple HIV risk behaviours and normative beliefs [20].

In relation to friendship networks, it was noted that they enable individuals at the community levels the ability to create opportunities to take preventive health actions and thereby promote effective uptake of health interventions in rural poor villages [30]. Similarly, in Karuga et al. [26] community health committee networks were seen to enhance community participation in health services. These networks (community health committee networks) were further observed to use their level of influence and resourcefulness in serving as back-ups to government in addressing PHC challenges at the community levels. The commonality among these networks is that they support information sharing at the local/community level, enabling easy reach to communities.

### Health facility-level networks

In relation to health facility-level networks, the review showed that these networks enable collaboration among PHC staff and also ensure the building of social capital that enhances accountability and access to community health services. The reviewed articles reported four facility-level networks located at the district level of care (Tables 3 and 4). These include professional advice networks [28], support/supervisory networks [21] and patient referral networks [18,24]. In the study by Sabot et al., [28]) where existing professional advice networks among healthcare workers in PHC units were examined, it was found that professional advice networks provide support for improvements in health provider practices than when health care workers are only given training (example, in-service training) without the involvement of their networks. These networks were further seen to provide support for healthcare professionals because they share expert knowledge on PHC issues and other resources by leveraging the capabilities of network members.

Similarly, it was observed that support/supervisory networks that draw on sympathetic cadres among PHC facility staff for support among ward-based outreach teams (WBOTs) in a rural South African district promote teamwork, problem-solving and participatory decision-making, and strengthen interpersonal relations among PHC providers [21]. The WBOTs are the teams responsible for the provision of preventive and promotive services at the community and household levels within a municipal ward. One of the reviewed articles [18] that explored the influence of patient referral networks on policy implementation showed that referral networks influence the treatment of childhood illness and non-communicable diseases by facilitating the sharing of patients among PHC facilities and higher levels of care as well as support patients from the community access to PHC facilities. Referral networks between women’s self-help groups (SHGs) and local health systems in India [24] were also noted to help in expanding relationships at village level, building the social capital that could lead to a more accountable and accessible community health delivery system Although the health facility-level networks (Table 4) were different, it is clear that they work towards a common goal of promoting/improving PHC implementation.

### Multi-partner networks that work across levels

The review further demonstrated that multi-partner networks that work across levels promote implementation by facilitating information and resource sharing, high professional trust and effective communication among actors (Tables 3 and 4). Five of the reviewed articles [22,23,25,27,29] reported on multi-partner networks, involving multiple partners working together to achieve a common goal (Tables 3 and 4). They included intersectoral collaboration networks [23,25], partnership networks [27], interorganisational networks [29] and communication networks [22]. The different names of the networks connote the objectives of the networks. Two of the articles reported on intersectoral collaboration networks [23,25]. In one of the articles that reported on intersectoral collaborations between organisations working on maternal and child health (MCH) and water and sanitation (WASH) in rural India [23], it was observed that when networks involve multiple partners, they enable growth of the organisations involved and also provide these organisations the opportunity to serve as gatekeepers of information. Similarly, with regard to mental health care, intersectoral collaboration networks were found to facilitate information and resource sharing among the involved organisations as means to strengthening people-centred care in Timor-Leste [25].

A partnership network was examined in one of the reviewed papers [27]. The specific focus was Mozambique’s Gavi-driven partnership network, which delivered human papillomavirus (HPV) vaccine during the demonstration phase to test a model for HPV vaccine delivery to girls aged between 9 and 13 years. The findings showed that this partnership network enabled high professional trust and also a favourable environment for leveraging partners’ capabilities.

In terms of inter-organisational networks, Ssengooba et al. [29] assessed organisations that support the provision of (1) HIV treatment, (2) maternal delivery services and (3) workforce strengthening. The study showed that the inter-organisational network enabled organisations to exhibit a broad range of functional roles in supporting the provision of the services. It was further noted that inter-organisational networks present opportunities to leverage faster communication and resource flow to boost the delivery of health services.

Communication networks were specifically examined by Kawonga et al. [22] who sought to understand the extent to which the health programme and district managers communicate in South Africa. The study revealed that communication networks enhance the flow of information among the programme managers. It also facilitates complex and varied interactions among programme and district managers.

## Discussion

This scoping review of papers reporting on the use of SNA in PHC implementation offers insights about actor networks and how they support PHC implementation in LMICs. It further provides evidence on the usefulness of SNA in HPA in understanding how networks support policy implementation and research gaps. In this regard, the review makes two main contributions to the field of health policy implementation and the value of SNA in health policy implementation work.

These are against the background that as it is common with scoping reviews, the literature search was not as methodologically rigorous as that for a systematic review. However, we ensured that only published peer-reviewed articles were included in the study. Also, the PRISMA-ScR [17] criteria were followed to guide the conduct and reporting of the review. We could not compare our findings to evidence in high-income countries as actor network studies in those countries were not PHC-focused and generally descriptive in nature [31].

First, the review provides evidence that, actor networks more likely play a role in PHC implementation in all LMICs. However, we could only obtain evidence for some countries. In these countries, actor networks play a role in PHC implementation across different levels of the health system. The review shows that networks support PHC implementation, offering empirical support for the role of networks highlighted in bottom-up policy implementation theory [10,11]. Our review also shows that networks support implementation at patient/household or community-level, health facility-level and through multi-partner networks that work across levels. In other words, patient/household or community-level networks could drive individual, households/families and community empowerment for increased social participation and enhanced self-care and self-reliance in health. Community empowerment and participation are known beneficial strategies for implementation of health services [32]. Moreover, effective community engagement is shown to positively impact on social capital that ultimately improves health status and reduces health inequalities [24]. For instance, patient/household or community-level networks could use their level of influence to help local implementers adapt PHC policy to local circumstances in ways that ensures broad policy and performance gains. Similarly, health facility-level networks could ensure comprehensive integrated PHC services, while multi-partner-level networks would enhance multi-sectoral policies and actions to address wider determinants of health. Existing evidence shows that integrated health services are key to achieving universal coverage [32,33] while multi-sectoral approaches to health has been identified as key for implementing health promotion and the prevention and control of diseases [33]. The evidence on SNA suggests that networks play an important role in these processes.

The review also reveals commonalities across the networks in terms of the way they support implementation. For example, the exchange of information among actors or implementers was seen across multiple networks. The review also shows that differences in networks tend to be driven by the context, goal and activities of a network. For instance, friendship network, tends to be driven by the context, goal and activities of close friends who do not live in same household but are comfortable to either turn to each other for advice, interest-free loan, or ask for help with harvest without paying [30], while peer networks, were driven by activities of people getting along or socialising regularly in a fixed location in predicting the susceptibility of individuals to good health behaviours [20,30]. This supports the argument of bottom-up theorists that the actors involved in policy implementation are influenced by the context, goals and activities of a policy. Therefore, in order to strengthen implementation, these components must be understood [12].

Bottom-up theory emphasises the discretionary power and critical influence of ‘policy implementors’ over policy implementation [12]. Our review shows the use of discretionary power either during the formation of a network or among the actors in the network. For instance, in the formation of a partnership network for HPV vaccine delivery, actors used their discretionary power to determine who they would partner with [27]. Additionally, for professional advice networks, health providers used their discretion to determine whether to seek or to give advice on the provision of antenatal, childbirth, postnatal and newborn care [26]. The consequences of these practices of discretionary power among actors within the networks could contribute to the commonalities or the differences observed but also demonstrate the significance for understanding networks in mitigating the implementation gap.

Our review further shows that SNA could be a useful tool in HPA, particularly in providing understanding of how networks influence policy implementation. In the reviewed papers, SNA was applied within different contexts and across different actors. SNA provided descriptions of the set of actors and members of actor networks, and characterised the relationships between the actors and the influence of these relationships. It was applied within several research designs and either as a stand-alone method or in combination with other relevant methods, implying the flexibility of SNA as a methodology. In the reviewed studies, SNA provided understanding of actors, context, content and processes of policy implementation, suggesting that this analytic approach offers value in generating policy relevant theoretical insights about networks and their influences on policy implementation.

The review also provides a foundation for such future primary, empirical research. To the best of our knowledge, this is the first scoping review on the role of actor networks in PHC implementation in LMICs. Given the very limited number of empirical studies identified, there is considerable scope for more research on the role of actor networks in PHC implementation, the paper therefore adds to the literature that documents actor networks and their role in the implementation of PHC. From the review, all networks were identified and defined within a particular context, however, there is limited understanding of the influence of the context on the networks and how the networks are managed in order to influence implementation.

## Conclusion

This body of literature we reviewed suggests that, actor networks exist across different levels and that they make a difference in PHC implementation. We argue that SNA could be a useful approach for health policy analysts looking at implementation. However, because of the limited body of evidence considered in this review, and the narrow range of country contexts covered in the included studies, we suggest it will be useful to further explore the use of SNA as part of HPA approach in implementation in a wider range of contexts.

## Paper context

Primary health care improvement is often undermined by implementation gaps in low- and middle-income countries. Limited attention has been paid to the role actor networks could play over the implementation. This body of literature reviewed suggests that, actor networks exist across different levels and that they make a difference in primary health care implementation. Social Network Analysis may be a useful approach to health policy analysis on implementation.

## References

[1] World Health Organization. Primary health care. Report of the international conference on primary health care, Alma-Ata, USSR. World Heal Organ; 1978.

[2] Adongo PB, Tapsoba P, Phillips JF, Tabong PTN, Stone A, Kuffour E, et al. The role of community-based health planning and services strategy in involving males in the provision of family planning services: a qualitative study in Southern Ghana. Reprod Health. 2013;10:1–12. 23890362 10.1186/1742-4755-10-36 PMC3726500

[3] Christopher J, May AL, Lewin S. Review of the impact of community health workers delivering curative interventions against malaria, pneumonia and diarrhoea on child mortality and morbidity in. Hum Resour Health. 2011;27:1–11. 10.1186/1478-4491-9-27 PMC3214180 22024435

[4] Bitton A, Fifield J, Ratcliffe H, Karlage A, Wang H, Veillard JH, et al. Primary healthcare system performance in low-income and middle-income countries: a scoping review of the evidence from 2010 to 2017. BMJ Glob Heal. 2019;4:e001551. 10.1136/bmjgh-2019-001551 PMC6703296 31478028

[5] Bitton A, Ratcliffe HL, Veillard JH, Kress DH, Barkley S, Kimball M, et al. Primary health care as a foundation for strengthening health systems in low- and middle-income countries. J Gen Intern Med. 2017;32:566–571. 27943038 10.1007/s11606-016-3898-5 PMC5400754

[6] Stenberg K, Hanssen O, Bertram M, Brindley C, Meshreky A, Barkley S, et al. Guide posts for investment in primary health care and projected resource needs in 67 low-income and middle-income countries: a modelling study. Lancet Glob Health [Internet]. 2019;7:e1500–10. 31564629 10.1016/S2214-109X(19)30416-4 PMC7024989

[7] World Health Organization. Primary health care. World Heal Organ [Internet]; 2019. Available from: https://www.who.int/news-room/fact-sheets/detail/primary-health-care

[8] Awoonor-Williams J, Tindana P, Dalinjong P, Nartey H, Akazili J. Does the operations of the National Health Insurance Scheme (NHIS) in Ghana align with the goals of primary health care? Perspectives of key stakeholders in northern Ghana. BMC Int Health Hum Rights [Internet]. 2016;16:1–11. 27576456 10.1186/s12914-016-0096-9 PMC5006541

[9] Dassah E, Aldersey HM, McColl MA, Davison C. Healthcare providers’ perspectives of providing primary healthcare services to persons with physical disabilities in rural Ghana. Prim Health Care Res Dev. 2019;20:20. 10.1017/S1463423619000495 PMC6609974 32799998

[10] Hanf K, Hjern B, Porter D. Local networks of manpower training in the federal republic of Germany and Sweden. In: Hanf K Scharpf FW, editors. Interorganizational policy making: limits to coordination and central control. London: Sage Publication; 1978.

[11] Hjern B, Porter DO. Implementation structures: a new unit of administrative analysis. Organ Stud. 1981;2:211–227.

[12] Barrett SM. Implementation studies: time for a revival? Personal reflections on 20 years of implementation studies. Public Adm. 2004;82:249–262.

[13] Elmore RE. Organizational models of social program implementation. Public Policy. 1978;26:185–22. 10308533

[14] Erasmus E, Orgill M, Schneider H, Gilson L. Mapping the existing body of health policy implementation research in lower income settings: what is covered and what are the gaps? Heal Policy Plan. 2014;29:iii35–50. 10.1093/heapol/czu063 25435535

[15] Arksey H, O’Malley L. Scoping studies: towards a methodological framework. Int J Soc Res Methodol Theory Pract. 2005;8:19–32.

[16] Levac D, Colquhoun H, O’Brien KK. Scoping studies: advancing the methodology. Implement Sci. 2010;5:1–9. 20854677 10.1186/1748-5908-5-69 PMC2954944

[17] Tricco AC, Lillie E, Zarin W, O’Brien KK, Colquhoun H, Levac D, et al. PRISMA extension for scoping reviews (PRISMA-ScR): checklist and explanation. Ann Intern Med. 2018;169:467–473. 30178033 10.7326/M18-0850

[18] Francetic I, Tediosi F, Kuwawenaruwa A. A network analysis of patient referrals in two district health systems in Tanzania. Health Policy Plan. 2020;36:1–14. 10.1093/heapol/czaa138 PMC7996649 33367559

[19] Mukong AK, Burns J. Social networks and antenatal care utilisation in Tanzania. Sci African [Internet]. 2020;9:e00535.

[20] Mulawa M, Yamanis TJ, Hill LM, Balvanz P, Kajula LJ, Maman S. Evidence of social network influence on multiple HIV risk behaviors and normative beliefs among young Tanzanian men. Soc Sci Med. 2016 Mar 1;153:35–43. 26874081 10.1016/j.socscimed.2016.02.002 PMC4788532

[21] Assegaai T, Schneider H. The supervisory relationships of community health workers in primary health care: social network analysis of ward-based outreach teams in Ngaka Modiri Molema District, South Africa. BMJ Glob Heal. 2019;4:1–9. 10.1136/bmjgh-2019-001839 PMC6936529 31908861

[22] Kawonga M, Blaauw D, Fonn S. Exploring the use of social network analysis to measure communication between disease programme and district managers at sub-national level in South Africa. Soc Sci Med. 2015;135:1–14. 25931377 10.1016/j.socscimed.2015.04.024

[23] Hoe C, Adhikari B, Glandon D, Das A, Kaur N, Gupta S. Using social network analysis to plan, promote and monitor intersectoral collaboration for health in rural India. PLoS ONE. 2019;14:1–12. 10.1371/journal.pone.0219786 PMC6636742 31314793

[24] Ruducha J, Hariharan D, Potter J, Ahmad D, Kumar S, Mohanan PS, et al. Measuring coordination between women’s self-help groups and local health systems in rural India: a social network analysis. BMJ Open. 2019;9:1–13. 10.1136/bmjopen-2019-028943 PMC6701569 31399457

[25] Hall T, Kakuma R, Palmer L, Minas H, Martins J, Armstrong G. Intersectoral collaboration for people-centred mental health care in timor-leste: a mixed-methods study using qualitative and social network analysis. Int J Ment Health Syst [Internet]. 2019;13:1–13. 31788024 10.1186/s13033-019-0328-1 PMC6858633

[26] Karuga RN, Kok M, Mbindyo P, Hilverda F, Otiso L, Kavoo D, et al. “It’s like these CHCs don’t exist, are they featured anywhere?”: social network analysis of community health committees in a rural and urban setting in Kenya. PLoS ONE. 2019;14:1–19. 10.1371/journal.pone.0220836 PMC6687128 31393923

[27] Soi C, Shearer J, Chilundo B, Muchanga V, Matsinhe L, Gimbel S, et al. Global health systems partnerships: a mixed methods analysis of Mozambique’s HPV vaccine delivery network actors. BMC Public Health. 2020;20:1–13. 32503479 10.1186/s12889-020-08958-1 PMC7275554

[28] Sabot K, Blanchet K, Berhanu D, Spicer N, Schellenberg J. Professional advice for primary healthcare workers in Ethiopia: a social network analysis. BMC Health Serv Res. 2020;20:1–16. 10.1186/s12913-020-05367-3 PMC7302001 32552727

[29] Ssengooba F, Kawooya V, Namakula J, Fustukian S. Application of social network analysis in the assessment of organization infrastructure for service delivery: a three district case study from post-conflict northern Uganda. Health Policy Plan. 2017;32:1193–1202. 28637228 10.1093/heapol/czx071 PMC5886158

[30] Chami GF, Ahnert SE, Voors MJ, Kontoleon AA. Social network analysis predicts health behaviours and self-reported health in African villages. PLoS ONE. 2014;9:e103500. 25072820 10.1371/journal.pone.0103500 PMC4114748

[31] Chambers D, Wilson P, Thompson C, Harden M. Social network analysis in healthcare settings: a systematic scoping review. PLoS ONE. 2012;7:e41911. 22870261 10.1371/journal.pone.0041911 PMC3411695

[32] Marston C, Hinton R, Kean S, Baral S, Ahuja A, Costello A, et al. Community participation for transformative action on women’s, children’s and adolescents’ health. Bull World Health Organ. 2016;94:376–382. 27152056 10.2471/BLT.15.168492 PMC4857226

[33] Narain J. Integrating services for noncommunicable diseases prevention and control: use of primary health care approach. Indian J Community Med. 2011;36:67–71. 10.4103/0970-0218.94712 PMC3354898 22628915

